# Combination Therapy With AOPT Intense Pulsed Light (Dual‐Band Vascular Filter) and 1064 nm Nd:YAG Laser for Solitary, Superficial Infantile Hemangioma: A Retrospective Study

**DOI:** 10.1111/jocd.70912

**Published:** 2026-05-14

**Authors:** Zhang Jiang, Peng Jiang, Feifeng Ran, Simin Li, Yuanyuan Xu, Yushuang Yang, Leifeng Liang, Li Yang, Changyuan Wei

**Affiliations:** ^1^ Departments of Breast Surgery Guangxi Medical University Cancer Hospital Nanning China; ^2^ Department of Plastic Surgery The Sixth Affiliated Hospital of Guangxi Medical University, the First People's Hospital of Yulin Yulin China; ^3^ Department of Oncology The Sixth Affiliated Hospital of Guangxi Medical University, the First People's Hospital of Yulin Yulin China; ^4^ Department of Medical Cosmetology The Sixth Affiliated Hospital of Guangxi Medical University, the First People's Hospital of Yulin Yulin China

**Keywords:** infantile hemangiomas, long‐pulsed laser, optimal pulse technology, therapeutic effect

## Abstract

**Objective:**

This study evaluated the efficacy and safety of a sequential protocol for treating solitary, superficial infantile hemangioma (IH). The protocol combined an Advanced Optimal Pulse Technology (AOPT) intense pulsed light system, which was equipped with a novel dual‐band vascular filter, with a subsequent long‐pulsed 1064 nm Nd:YAG laser. We compared this sequential approach to treatment with a long‐pulsed Nd:YAG laser alone.

**Methods:**

In this retrospective cohort study, 257 treatment‐naïve children with solitary, superficial IH were included. Patients were divided into two groups based on a chronological cutoff following the introduction of a new AOPT intense pulsed light device in March 2024. The combination therapy group (*n* = 113) received treatment with the AOPT intense pulsed light system immediately followed by the Nd:YAG laser. The monotherapy group (*n* = 144) was treated with the Nd:YAG laser alone. The primary efficacy endpoint was the overall improvement rate. Time to improvement was analyzed using Cox proportional hazards regression, and safety was assessed by the incidence of adverse events.

**Results:**

The combination therapy group achieved a significantly higher overall response rate (92.9% vs. 82.6%, *p* = 0.014). Cox regression analysis confirmed that combination therapy significantly accelerated the time to improvement (hazard ratio = 1.43, 95% CI: 1.08–1.88, *p* = 0.011). The final complete cure rate and the overall incidence of adverse events were comparable between the two groups.

**Conclusion:**

For solitary, superficial IH, the sequential treatment protocol using an AOPT intense pulsed light system with an integrated dual‐band vascular filter, followed by long‐pulsed Nd:YAG laser, is a safe and effective local strategy. It significantly accelerates lesion regression and improves the treatment response rate compared to Nd:YAG laser monotherapy.

## 1 Introduction

Infantile hemangiomas (IH) are among the most common benign vascular tumors in childhood, with an estimated incidence ranging from 4% to 5% [1]. They typically manifest at birth or within the first postnatal month. While the head, face, and extremities represent the most frequently involved superficial sites, IH can also involve deeper or more critical structures, including mucous membranes, muscle, bone, and even intracranial locations [1, 2]. During the proliferative phase, the lesion undergoes rapid expansion, which may be complicated by necrosis and ulceration. Therefore, early intervention has become a well‐established consensus to mitigate these risks and improve clinical outcomes [3].

The optimal treatment for solitary, superficial IH remains a subject of debate. Conventional therapies are each limited by drawbacks. Systemic medications, such as oral propranolol, risk overtreatment and can induce side effects or drug resistance [4, 5]. Common adverse effects include sleep disturbances, peripheral coldness, and agitation; more serious but less frequent events, such as bradycardia, hypotension, bronchospasm, and hypoglycemia‐related seizures, may necessitate dose adjustment or treatment discontinuation [6]. These potential risks frequently heighten parental concern and can compromise treatment adherence. Surgical intervention tends to leave scars, while traditional cryotherapy and radioisotope applicator therapy are associated with relatively significant side effects [7, 8]. Given these limitations, non‐invasive laser modalities remain a valuable therapeutic option for such lesions, even considering their higher cost and the frequent need for multiple treatment sessions [9].

The long‐pulsed 1064 nm Nd:YAG laser can effectively target vascular structures in deeper tissue layers and has become an important tool for treating vascular lesions [10, 11, 12]. It is also considered a safe and effective modality for IH [13]. However, its efficacy as a monotherapy appears inferior to that achieved in combination with the pulsed dye laser (PDL). This difference likely stems from their complementary mechanisms of action on vessels at different depths [14].

The theory of selective photothermolysis, as proposed by Anderson, indicates that oxyhemoglobin exhibits high selective absorption peaks at short wavelengths of 418 nm, 542 nm, and 577 nm [15]. Intense Pulsed Light (IPL) emits a broad spectrum of light that includes the specific absorption peaks of hemoglobin, enabling effective photothermal coagulation of superficial blood vessels. This mechanism underpins its proven efficacy in treating superficial IH [16]. Advanced Optimal Pulse Technology (AOPT) is an enhanced form of IPL that enables more precise energy delivery via independent sub‐pulse modulation. AOPT‐based IPL (AOPT‐IPL) system, equipped with interchangeable filters [17], has long been extensively utilized in facial rejuvenation [18, 19], and the treatment of meibomian gland dysfunction [20]. A study has demonstrated that AOPT‐IPL therapy can achieve satisfactory clinical outcomes in the treatment of skin aging with prominent vascular abnormalities [21]. This study employed an AOPT‐IPL fitted with a dual‐band vascular filter. This filter is designed for vascular lesion treatment, optimized from the original single‐band structure to a targeted dual‐band configuration (530–650 nm and 900–1200 nm). The shorter band (530–650 nm) targets superficial to middle capillary plexuses by matching hemoglobin's primary absorption peaks (542 nm and 577 nm) while minimizing melanin competition [22]. The longer band (900–1200 nm) penetrates more deeply to target secondary absorption peaks and affect deeper vessels [23]. While this dual‐band technology (400–600 nm and 800–1200 nm) has demonstrated reliable clinical safety and efficacy in the treatment of acne [24, 25], its application using the dual‐band vascular filter for infantile hemangioma has not been previously reported.

These two modalities combine to provide an ideal complementary therapeutic effect for IH. However, the efficacy and safety of sequentially combining an AOPT‐IPL equipped with a dual‐band vascular filter and a long‐pulsed 1064 nm Nd:YAG laser for IH remain unknown. To evaluate the clinical utility of this protocol, we performed a retrospective study to assess its outcomes.

## 2 Materials and Methods

### 2.1 Clinical Data

#### 2.1.1 Study Participants

This single‐center retrospective study included 257 treatment‐naive children with solitary, superficial IH, treated at our hospital's outpatient department between June 2022 and June 2025. The cohort consisted of 102 males and 155 females. Based on a chronological cutoff following the introduction of a new AOPT‐IPL device in March 2024, patients treated before March 2024 received monotherapy, while those treated in or after March 2024 received combination therapy. The study protocol received approval from the institutional ethics committee of The First People's Hospital of Yulin, Guangxi (Approval No. YLSY‐IRB‐SR‐2026006). Written informed consent was obtained from all participants' legal guardians. Additional specific consent was acquired for the publication of clinical photographs.

#### 2.1.2 Inclusion and Exclusion Criteria

##### 2.1.2.1 Inclusion Criteria

1. Age at first treatment ≤ 12 months;
2. Diagnosis of a solitary, superficial IH confirmed by clinical visual inspection, palpation, and ultrasonography (B‐scan);
3. The lesion was treatment‐naïve;
4. The overlying skin of the lesion was intact, without ulceration or infection.

##### 2.1.2.2 Exclusion Criteria

1. Lesions showing signs of spontaneous regression (e.g., softening in texture and fading in color) at presentation;
2. Diagnosis of a deep or mixed‐type hemangioma or any form of vascular malformation (e.g., venous malformation);
3. Any history of prior treatment for the target lesion (e.g., sclerotherapy, laser therapy, cryotherapy, and topical medication).

### 2.2 Instruments and Equipment

#### 2.2.1 Long‐Pulsed 1064 nm Nd:YAG Laser

Treatments were delivered using a Lumenis One multi‐application laser platform (Lumenis Ltd., USA). The system operated at a wavelength of 1064 nm with a spot size of 6 mm or 9 mm, chosen according to the lesion's size and location. We employed a single‐, double‐, or triple‐pulse mode. Fluence settings ranged from 45 to 120 J/cm^2^, and pulse duration was set between 2 and 20 ms. The handpiece featured integrated skin cooling.

#### 2.2.2 Advanced Optimal Pulse Technology Intense Pulsed Light Treatment

For IPL treatment, we used a Strong Light Star AOPT‐IPL system (Jilin Province Kinglaser Laser Co. Ltd., China). A dedicated vascular filter with a targeted dual‐band design (530–650 nm and 900–1200 nm) was applied for its suitability across various vascular lesions. The fluence ranged from 12 to 25 J/cm^2^. Dual‐pulse or triple‐pulse mode was applied. Pulse width was configured between 3.5 and 5.5 ms, with an inter‐pulse delay of 20 to 50 ms. This handpiece also incorporated integrated skin cooling.

### 2.3 Pretreatment Preparation

The treatment area was cleansed and shaved. A detailed medical record was established, documenting the lesion's size, color, surface temperature, and blanchability (or capillary refill). Standardized digital photographs were taken before and after each treatment session for efficacy comparison. Patients who had taken any photosensitizing agents within the preceding 2 weeks were excluded from the treatment session.

### 2.4 Treatment Protocol and Endpoint

#### 2.4.1 Definition of Therapeutic Endpoint

The therapeutic endpoint was defined as the minimal dose required to achieve shrinkage, depression, color change to grayish‐white or dark red, and hardening of the hemangioma through single or multiple irradiations with laser or AOPT‐IPL, while minimizing impact on the surrounding skin and systemic effects.

#### 2.4.2 Treatment Procedures

Both the child and operator wore appropriate laser safety goggles or protective eyewear. For periorbital and eyelid hemangiomas, an opaque titanium corneal eye shield was placed. The treatment area was routinely disinfected, and a clear chilled light‐conducting gel was applied as a coupling agent:

- Combination therapy group

Treatment was initiated with the AOPT‐IPL system using the dedicated vascular filter. Parameters were selected based on lesion characteristics (location, size, color, and turgor). For bright red, high‐turgor lesions, we employed a relatively lower fluence with longer pulse durations; for darker red, low‐turgor lesions, we applied a higher fluence with standard or shorter pulse durations. Specific parameters were: fluence 12–25 J/cm^2^, pulse duration 3.5–5.5 ms, typically delivering 2–3 pulses with an inter‐pulse delay of 20–50 ms. IPL treatment was continued until approximately 50% of the lesion area exhibited the desired endpoint response (e.g., color change to dark red or grayish‐white). Immediately thereafter, treatment was switched to the long‐pulsed Nd:YAG laser and continued until the entire lesion fully met the therapeutic endpoint. In all subsequent treatment sessions, parameters were individually adjusted based on the response observed from the previous session.

b Monotherapy group

Treatment was conducted using the long‐pulsed 1064 nm Nd:YAG laser alone. A spot size of 6 mm or 9 mm was chosen according to the lesion size, with a fluence range of 45–120 J/cm^2^. The therapeutic endpoint and the principle of parameter adjustment in follow‐up sessions were identical to the laser phase described for the Combination Therapy Group.

### 2.5 Treatment Interval and Post‐Treatment Care

Following each treatment, the lesion area was cooled with an ice pack for at least 30 min. Parents were instructed to keep the area clean, avoid direct sunlight for 3–5 days, and refrain from using hot water on the site. A follow‐up evaluation and subsequent treatment session were scheduled every 3–4 weeks. Effective sun protection was recommended for 1 month after each laser treatment.

All parents were informed of potential side effects, including swelling, erythema, blistering, skin erosion, and others. Specific guidance was provided to apply aureomycin ointment or mupirocin ointment twice daily in case blistering or erosion occurred.

### 2.6 Patient Pain Management and Cooperation

For analgesia, all patients received non‐pharmacological pain control using integrated handpiece cooling and post‐procedural cold packs. Sedation was not administered routinely. Most infants tolerated the procedure well with parental holding and distraction. For infants who could not be comforted, gentle positioning was performed by a parent under physician assistance. In rare cases where positioning alone was inadequate to ensure safe treatment, oral sedation with 10% chloral hydrate (50 mg/kg) was used as a rescue intervention. Notably, continuous pain control via cooling was maintained even when sedation was required.

### 2.7 Criteria for Evaluating Therapeutic Effect

Three experienced physicians independently assessed clinical outcomes, with one assessor blinded to treatment allocation. The primary criterion was the estimated percentage reduction in total lesion volume. Descriptions from the Achauer scoring system [26] regarding improvements in color, size, thickness, and texture provided supplementary reference for a comprehensive judgment. Improvement was classified into four grades: Grade I (No Improvement, 0%–25%), Grade II (Mild Improvement, 26%–50%), Grade III (Moderate Improvement, 51%–75%), and Grade IV (Marked Improvement, 76%–100%). In cases of initial disagreement, the three assessors jointly reviewed the images to reach a consensus. Efficacy was evaluated 1 month after the fourth treatment session if the total course exceeded 4 months, with a final evaluation 1 month after the last treatment. For treatment courses not exceeding 4 months, evaluation was performed 1 month after the final session.

In this study, the overall response rate (ORR) was defined as the percentage of patients achieving Grade III or IV, while the complete cure rate represented the percentage of patients meeting the “Complete Cure” criteria. “Complete Cure” was defined as meeting the following criteria 1 month after the final treatment: (1) Color Doppler ultrasound confirmation of absent abnormal blood flow signals and complete resolution of the abnormal vascular nest in the original lesion area; (2) Achievement of the highest improvement grade (Grade IV) with an actual volume reduction of ≥ 95%.

### 2.8 Safety Assessment

Adverse events related to treatment were actively monitored and recorded for all children from the first intervention until 1 month after the final session. These events were categorized primarily by clinical manifestation as blistering, crusting, erosion, significant edema, pigmentary changes (hyperpigmentation or hypopigmentation), hemorrhage, necrosis, or infection. The primary safety endpoint was the overall incidence of adverse reactions, defined as the percentage of children experiencing at least one adverse event of any type or grade.

### 2.9 Follow‐Up

Follow‐up was divided into two tiers. Prespecified follow‐up: All patients with complete cure (no flow on ultrasound and ≥ 95% clinical improvement) underwent a prespecified ultrasound at 3 months post‐cure to screen for recurrence, and all completed this follow‐up. For patients without complete cure, the last treatment assessment was used as the study endpoint. Recommended follow‐up: After the 3‐month prespecified visit, patients were advised to return every 3 months for up to 1 year to monitor long‐term outcomes. Additional follow‐up time was calculated from the 3‐month post‐cure time point.

### 2.10 Statistical Analysis

Data management and organization were performed using Microsoft Office Excel 2016. Statistical analyses were conducted with SPSS (version 24.0) and R software (version 4.5.2). Normally distributed measurement data are presented as mean (*x̄* ± *s*) standard deviation, with between‐group comparisons performed using the independent samples *t*‐test for two groups or one‐way analysis of variance for multiple groups. Non‐normally distributed measurement data and ordinal data were analyzed using non‐parametric rank sum tests, specifically the Mann–Whitney U test or the Kruskal–Wallis H test. Categorical data are presented as number, with between‐group comparisons performed using the chi‐square test or Fisher's exact test. The cumulative improvement rate for efficacy was analyzed using the Cox proportional hazards regression model. All tests were two‐sided, and a *p*‐value of < 0.05 was considered statistically significant. To verify the robustness of the primary Cox regression model, we employed three sensitivity analyses. First, an *E*‐value analysis quantified the potential bias from unmeasured confounding [27, 28]. Second, bootstrap resampling with 1000 replicates assessed the stability of the regression coefficients [29, 30]. Finally, we performed cross‐validation using a discrete‐time logit model to account for the discrete nature of time defined by treatment sessions [31, 32].

## 3 Results

### 3.1 Baseline Characteristics

A total of 257 children with superficial IH who met the eligibility criteria were retrospectively included and categorized into two groups based on the treatment received: the Monotherapy Group (long‐pulsed laser alone, *n* = 144) and the Combination Therapy Group (AOPT‐IPL combined with long‐pulsed laser, *n* = 113). The baseline demographic and clinical characteristics of the two groups are summarized in Table 1.

**TABLE 1:** Baseline clinical characteristics of patients with hemangioma.

Characteristic	Monotherapy group (*n* = 144)	Combination therapy group (*n* = 113)	Statistic	*p*
*Gender, n (%)*				
Male	57 (39.6%)	45 (39.8%)	*χ* ^*2*^ = 0.002	0.969
Female	87 (60.4%)	68 (60.2%)		
*Preterm infant, n (%)*				
Yes	7 (4.9%)	8 (7.1%)	*χ* ^*2*^ = 0.567	0.451
No	137 (95.1%)	105 (92.9%)		
*Age at treatment initiation (months)*	3.0 (2.0–5.0)	4.0 (2.0–5.5)	*Z =* −0.431	0.666
*Fitzpatrick skin phototype* ^a^, *n (%)*				
Type III	86 (59.7%)	69 (61.1%)	*χ* ^*2*^ = 0.047	0.828
Type IV	58 (40.3%)	44 (38.9%)		
*Lesion location, n (%)*				
Head/Neck	62 (43.1%)	40 (35.4%)	*χ* ^*2*^ = 2.183	0.535
Trunk	40 (27.8%)	34 (30.1%)		
Limbs	36 (25.0%)	31 (27.4%)		
Perineum/Genitalia/Buttocks	6 (4.2%)	8 (7.1%)		
*Maximum lesion diameter (cm), n (%)*				
≤ 1 cm	56 (38.9%)	40 (35.4%)	*Z =* −1.014	0.311
1 cm < Diameter ≤ 2 cm	38 (26.4%)	24 (21.2%)		
2 cm < Diameter ≤ 4 cm	36 (25.0%)	37 (32.7%)		
> 4 cm	14 (9.7%)	12 (10.6%)		

*Note:* Data are presented as number (%) or median (interquartile range). Categorical variables (e.g., gender and lesion location) were compared using the Chi‐square test. Continuous variables (e.g., age at treatment initiation), which did not follow a normal distribution, as well as the ordinal variable “maximum lesion diameter”, were compared using the Mann–Whitney U test (reported as *Z* value).

^a^ Only Fitzpatrick skin phototypes III and IV were present in this study cohort. *p* < 0.05 was considered statistically significant.

The two groups were well‐balanced at baseline. There were no statistically significant differences in gender distribution, proportion of preterm births, median age at treatment initiation, Fitzpatrick skin phototype, lesion location, or maximum lesion diameter (all *p* > 0.05, Table 1).

### 3.2 Efficacy Analysis: Combination Therapy vs. Monotherapy

The distribution of improvement grades and primary efficacy endpoints are summarized in Tables 2 and 3, respectively. The Combination Therapy Group demonstrated significant advantages in key efficacy indicators.

**TABLE 2:** Distribution of improvement grades in the two groups.

Item	Monotherapy group (*n* = 144)	Combination therapy group (*n* = 113)
*After the 4th treatment sessions*		
Grade I (no improvement)	13 (9.0%)	2 (1.8%)
Grade II (mild improvement)	21 (14.6%)	10 (8.8%)
Grade III (moderate improvement)	32 (22.2%)	18 (15.9%)
Grade IV (marked improvement)	78 (54.2%)	83 (73.5%)
*After all treatment sessions (final)*		
Grade I (no improvement)	11 (7.6%)	2 (1.8%)
Grade II (mild improvement)	14 (9.7%)	6 (5.3%)
Grade III (moderate improvement)	14 (9.7%)	14 (12.4%)
Grade IV (marked improvement)	105 (72.9%)	91 (80.5%)

**TABLE 3:** Comparison of primary efficacy endpoints between the two groups.

Primary efficacy endpoints	Monotherapy group (*n* = 144)	Combination therapy group (*n* = 113)	Statistic	*p*
Overall response rate (Grade III + IV)	119 (82.6%)	105 (92.9%)	*χ* ^*2*^ = 5.980	0.014
Complete cure rate	106 (73.6%)	86 (76.1%)	*χ* ^*2*^ = 0.209	0.648
*Evaluation after the 4th treatment sessions*				
Overall response rate after 4 sessions (Grade III + IV)	110 (76.4%)	101 (89.4%)	*χ* ^*2*^ = 7.272	0.007
Complete cure rate after 4 sessions	63 (43.8%)	51 (45.1%)	*χ* ^*2*^ = 0.049	0.825

*Note:* The overall improvement rate includes patients with Grade III or IV improvement. Complete cure requires meeting both ultrasound‐confirmed absence of abnormal blood flow and clinical criteria (Grade IV, ≥ 95% improvement). Chi‐square test was used. *p* < 0.05 is significant.

**FIGURE 1:**
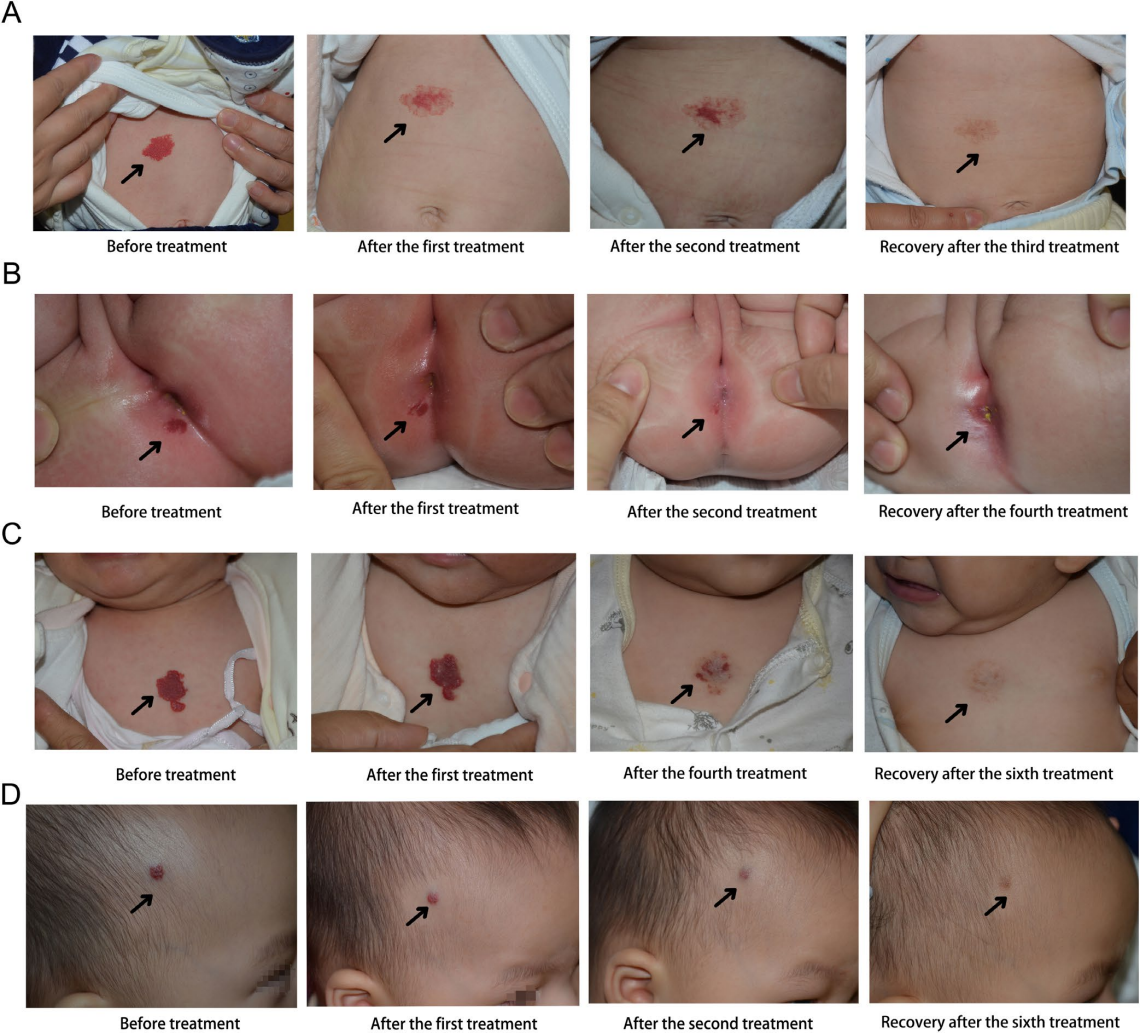
Typical Clinical Course of Infantile Hemangiomas under Different Treatment Regimens (A) A 2‐month‐old female with an abdominal IH achieved complete regression of the lesion after three treatment cycles with AOPT‐IPL combined with sequential long‐pulsed Nd:YAG laser therapy. (B) A 2‐month‐old female with a perianal IH achieved complete regression after four treatment cycles with the combination therapy. (C) A 4‐month‐old male with a chest wall IH achieved complete regression after six treatment cycles with monotherapy using the long‐pulsed Nd:YAG laser alone. (D) A 6‐month‐old male with a scalp IH achieved complete regression after six treatment cycles with the combination therapy.

Statistical analysis revealed that the ORR (Grade III or IV) was significantly higher in the Combination Therapy Group than in the Monotherapy Group (92.9% vs. 82.6%, *p* = 0.014). However, the final complete cure rate (ultrasound‐confirmed absence of abnormal blood flow, ≥ 95% improvement in Grade IV) did not differ significantly between the two groups (76.1% vs. 73.6%, *p* = 0.648). Similarly, the ORR after the 4th treatment session was significantly higher in the Combination Therapy Group (89.4% vs. 76.4%, *p* = 0.007), yet the cure rate at this interim time point showed no significant difference (45.1% vs. 43.8%, *p* = 0.825). Regarding the distribution of improvement grades, the proportion of patients achieving Grade IV (marked improvement) was higher in the Combination Therapy Group, both at the early treatment stage (73.5% vs. 54.2%) and at the final follow‐up (80.5% vs. 72.9%). Representative clinical photographs of the treated children are shown in Figure 1.

### 3.3 Analysis of Time to Improvement and Influencing Factors

**FIGURE 2:**
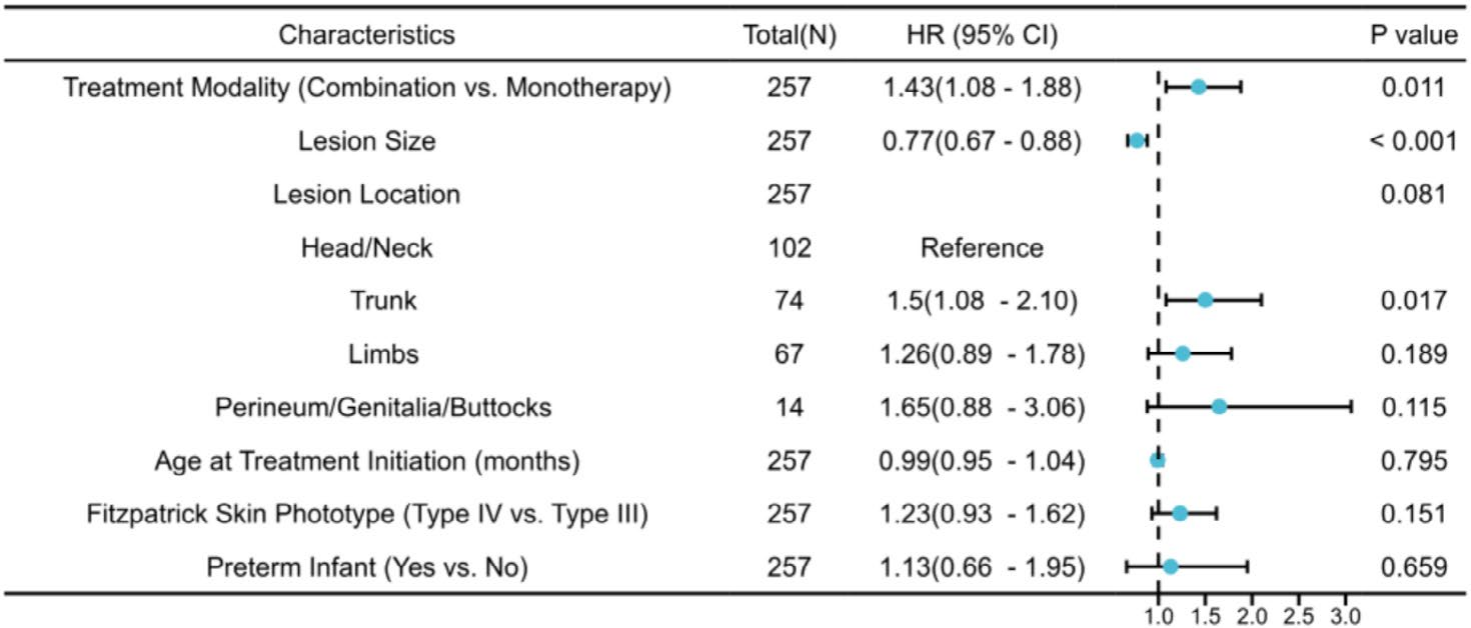
Forest plot of the Cox proportional hazards regression analysis for factors associated with time to significant improvement in infantile hemangiomas. Note: The plot shows HR with 95% CI and corresponding *p* values for each clinical characteristic among 257 enrolled patients. The vertical dashed line indicates the null effect threshold of HR = 1. CI, confidence interval; HR, hazard ratio.

The Cox proportional hazards regression model was employed to assess the independent effect of the treatment modality on the time to overall response (achieving Grade III or IV) of IH. Variable selection was based primarily on clinical relevance and informed by previous literature [33, 34]. The final multivariable model incorporated the core study variable of treatment modality, established strong prognostic factors (lesion size, location, age at initial treatment, and history of prematurity), and Fitzpatrick skin phototype [35], which correlates with laser treatment efficacy. Variables such as gender were excluded, as they showed no significant association in univariate analysis (all *p* > 0 0.05) and currently lack published evidence as strong predictors of hemangioma treatment response. These results are detailed in Table 4 and Figure 2.

**TABLE 4:** Results of Cox proportional hazards regression analysis for factors influencing the time to significant improvement in hemangioma.

Variable	Adjusted hazard ratio (HR)	95% confidence interval	*p*
Treatment modality (combination vs. monotherapy)	1.43	1.08–1.88	0.011
Lesion size	0.77	0.67–0.88	< 0.001
Lesion location			0.081
Trunk vs. Head/Neck	1.50	1.08–2.10	0.017
Limbs vs. Head/Neck	1.26	0.89–1.78	0.189
Perineum/Genitalia/Buttocks vs. Head/Neck	1.65	0.88–3.06	0.115
Age at treatment initiation (months)	0.99	0.95–1.04	0.795
Fitzpatrick skin phototype (Type IV vs. Type III)	1.23	0.93–1.62	0.151
Preterm infant (yes vs. no)	1.13	0.66–1.95	0.659

*Note:* The Cox proportional hazards regression model was adjusted for all variables listed in the table. Hazard ratios (HRs) are presented for the first group relative to the reference group (listed after “vs.”). An HR > 1 indicates a favorable factor for faster cure (accelerated cure), while an HR < 1 indicates an unfavorable factor (delayed cure). The treatment modality uses the “Monotherapy Group” as the reference. Lesion size was analyzed as a continuous variable. *p* < 0.05 is significant.

Sensitivity analyses verified the robustness of our primary findings. The *E*‐value (point estimate 2.21, lower limit 1.37) showed that substantial unmeasured confounding would be required to negate the observed treatment effect (HR = 1.43). Bootstrap resampling (1000 replicates) confirmed stable regression coefficients (bias‐corrected 95% CI 0.101–0.600, *p* = 0.002, minimal bias). Discrete‐time logit analysis yielded results consistent with the primary Cox model (OR = 1.69, 95% CI 1.23–2.33, *p* = 0.001), with good model fit confirmed by the Hosmer–Lemeshow test (*p* = 0.079).

After adjusting for age at treatment initiation, lesion location, and size, Cox regression identified combination therapy as an independent factor associated with accelerated improvement (HR = 1.43, 95% CI: 1.08–1.88, *p* = 0.011). Thus, patients receiving combination therapy reached the improvement endpoint 1.43 times faster than those on monotherapy. Lesion size also emerged as a significant prognostic factor, where larger lesions correlated with a slower rate of improvement (HR = 0.77, *p* < 0.001).

### 3.4 Safety Outcomes

The safety analysis results are presented in Table 5. The overall incidence of adverse events was 27.4% (31/113) in the Combination Therapy Group and 24.3% (35/144) in the Monotherapy Group, a difference that was not statistically significant (*χ* ^*2*^ = 0.929, *p* = 0.335). Pigmentary changes were the most common adverse event in the Monotherapy Group, occurring in 15.3% (22/144) of patients. This event was less frequent in the Combination Therapy Group, with an incidence of 9.7% (11/113). Rates of crusting (10.6% vs. 6.9%), erosion (8.0% vs. 4.2%), and significant edema (3.5% vs. 0.7%) were numerically higher with combination therapy. Necrosis was reported in a small number of cases in both groups (4.4% and 3.5%, respectively). Infection occurred only in the Combination Therapy Group (1.8%), with no instances observed in the Monotherapy Group. All adverse events except post‐inflammatory hyperpigmentation resolved with symptomatic management. No patient discontinued treatment due to an adverse event, and all hyperpigmentation cases demonstrated a gradual fading trend during the 3‐month follow‐up.

**TABLE 5:** Comparison of treatment‐related adverse events between the two groups.

Adverse event	Monotherapy group (*n* = 144)	Combination therapy group (*n* = 113)
Overall incidence, *n* (%)	35 (24.3%)	31 (27.4%)
Comparison (*χ* ^*2*^ test)	*χ* ^*2*^ = 0.929	*p* = 0.335
*Common adverse events, n* (%)		
Blistering	9 (6.3%)	10 (8.8%)
Crusting	10 (6.9%)	12 (10.6%)
Erosion	6 (4.2%)	9 (8.0%)
Significant edema	1 (0.7%)	4 (3.5%)
Pigmentary changes	22 (15.3%)	11 (9.7%)
Hemorrhage	0 (0.0%)	1 (0.9%)
Serious adverse events, *n* (%)	5 (3.5%)	7 (6.2%)
Necrosis	5 (3.5%)	5 (4.4%)
Infection	0 (0.0%)	2 (1.8%)

*Note:* Data are presented as the number of cases with the adverse event (percentage of the total group). All incidence rates refer to the proportion of patients. Serious adverse events were defined in this study as those potentially leading to permanent damage or requiring active medical intervention, specifically necrosis and infection. *p* < 0.05 is significant.

### 3.5 Long‐Term Follow‐Up Outcomes Beyond 3 Months

Among patients who achieved complete cure, 62 patients completed the recommended long‐term follow‐up (exceeding the 3‐month prespecified post‐treatment visit) and were evaluated for additional follow‐up duration, recurrence, and cosmetic outcomes. These findings are summarized in Table 6.

**TABLE 6:** Summary of recommended long‐term follow‐up (> 3 months after confirmation of complete cure).

Treatment group	*n*	Additional follow‐up (months)	Range (months)	Recurrence (*n*, %)	Presentation	Cosmetic outcome^a^
Monotherapy	32	Median: 5 [IQR: 3–8] Mean ± SD: 5.8 ± 3.2	1–16	1 (3.1%)	Punctate blood flow signals on ultrasound	No change from 3‐month
Combination therapy	30	Median: 5 [IQR: 3–8] Mean ± SD: 6.2 ± 4.9	1–18	1 (3.3%)	Punctate blood flow signals on ultrasound	No change from 3‐month

*Note:* Data are presented as median [IQR] or mean ± SD.

Abbreviations: IQR, interquartile range (25th to 75th percentile); SD, standard deviation.

^a^ At the prespecified 3‐month follow‐up, common adverse events (blistering, crusting, erosion, significant edema, pigmentary changes, and hemorrhage) occurred in 6 patients in the combination group and 5 in the monotherapy group. No serious adverse events (necrosis and infection) were observed in either group. During extended follow‐up, no patient experienced worsening of these events or developed new serious adverse events. Therefore, “No change from 3‐month” indicates that long‐term cosmetic outcomes remained stable relative to the prespecified 3‐month assessment.

Recurrence rates remained low in both treatment groups, with one recurrent case detected in each group (monotherapy: 3.1%, combination therapy: 3.3%). In the monotherapy group, the recurrent case presented with punctate blood flow signals on ultrasound at 5 months of additional follow‐up after 7 primary treatment sessions. The patient received one supplementary laser session, with no abnormal ultrasound findings at 3 months after retreatment (total additional follow‐up: 8 months). In the combination therapy group, the recurrent case showed punctate blood flow signals on ultrasound at 3 months of additional follow‐up after 3 primary treatment sessions. This patient also underwent one supplementary laser session, with no residual abnormalities detected at 3 months post‐retreatment (total additional follow‐up: 6 months).

All patients exhibited stable cosmetic outcomes, with no noticeable differences compared with the 3‐month prespecified follow‐up assessment. All recurrent lesions were successfully controlled with a single additional treatment session.

## 4 Discussion

Although previous studies have also investigated the combination of 1064 nm Nd:YAG laser with other therapies for IH, they have predominantly focused on PDL [36] or combination regimens of laser with medications such as propranolol [4, 37]. Meanwhile, large‐scale studies of laser‐only treatment specifically for the “solitary, superficial IH” subpopulation remain scarce. This retrospective study analyzed the largest cohort to date of patients with solitary, superficial IH treated exclusively with either a combined regimen of 1064 nm Nd:YAG laser and IPL or with 1064 nm Nd:YAG laser monotherapy.

Evidence from clinical practice indicates that monotherapy with either IPL or the 1064 nm laser for IH leaves room for improvement in terms of efficacy [38, 39]. The innovative dual‐band vascular filter of our AOPT‐IPL system differentiates our protocol from conventional broad‐spectrum IPL treatments. Unlike the widely used 590 nm cutoff filter (590–1200 nm) in prior IH research [39, 40], our dual‐band filter (530–650 nm and 900–1200 nm) precisely targets the 542 nm and 577 nm hemoglobin peaks with minimal melanin competition. Therefore, we hypothesized that pretreatment with a novel, AOPT‐IPL protocol, followed sequentially by a long‐pulsed Nd:YAG laser, could overcome the limitations of monotherapy through synergy. This study confirmed our hypothesis, as the combination therapy regimen produced a significantly higher final overall improvement rate than monotherapy. More importantly, analysis using a Cox proportional hazards regression model showed that combination therapy significantly accelerated the improvement process, increasing the rate of achieving improvement by a factor of 1.43 relative to the Monotherapy Group. The observation of a higher improvement rate without a difference in cure rate can be interpreted as follows: the AOPT‐IPL pretreatment in the combination protocol rapidly and effectively eliminates superficial vascular components, leading to significant lightening of lesion color and an initial reduction in volume, which manifests as a higher objective improvement rate early in the treatment course. In contrast, the deeper vascular nests often require multiple subsequent Nd:YAG laser sessions for complete coagulation and absorption. Thus, while the time to reach the final improvement endpoint is shortened, the ultimate complete clearance rates may converge with longer follow‐up, highlighting the core advantage of combination therapy in accelerating disease control and shortening the overall clinical course. The superiority of the combination therapy was evident early in the treatment protocol (before the fourth session), indicating its capacity to control the condition sooner, reduce treatment duration, and thereby alleviate the burden on families. The underlying mechanism may involve the dual‐band energy of the AOPT‐IPL gently acting on superficial‐to‐mid dermal vessels, inducing pre‐contraction and a photothermal priming effect that could optimize the energy deposition and efficacy of the subsequent Nd:YAG laser on deeper target vessels [41]. This study also reaffirmed that larger lesion size is an independent prognostic factor for a slower improvement rate, providing crucial guidance for formulating individualized treatment plans.

Regarding safety, the lack of a statistically significant difference in overall adverse events between the combination and Monotherapy Groups likely stems from several considerations. As a broadband source, IPL emits a spectrum that includes wavelengths absorbed not only by hemoglobin but also by epidermal melanin. In contrast to the single‐wavelength 1064 nm Nd:YAG laser, which operates where melanin absorption is lower, IPL treatment is more prone to competitive energy absorption by melanin. This absorption may partly contribute to adverse effects, counterbalancing the theoretical safety benefit of its targeted design [35, 42, 43]; moreover, parameter optimization in combination therapy is more intricate and requires greater operator skill. Therefore, the absence of a significant safety disadvantage for the combined regimen indicates that, with standardized and proficient application, the potential added risks of IPL can be adequately managed to achieve a safety outcome comparable to established laser monotherapy.

This study has several limitations. First, although multivariate adjustments were applied, the retrospective design may still be susceptible to residual confounding factors. Second, despite employing a blinded evaluation process following a standardized protocol, the assessment of efficacy retains a degree of subjectivity. Third, the sample size may be underpowered to detect efficacy differences within certain specific subgroups, such as those based on different anatomical locations. Fourth, the prespecified follow‐up period was only 3 months after treatment, and the recommended long‐term follow‐up had a high loss‐to‐follow‐up rate (only 24.1% of patients completed), which may introduce bias in evaluating recurrence and long‐term cosmetic outcomes. Future prospective, randomized controlled trials with larger cohorts and longer follow‐up are warranted to further validate its long‐term efficacy, safety, and health‐economic value [44, 45].

## 5 Conclusion

This study confirms that for solitary, superficial IH, a sequential protocol using an AOPT‐IPL system (featuring a dual‐band vascular filter) followed by long‐pulsed 1064 nm Nd:YAG laser significantly accelerates lesion regression while enhancing the improvement rate, compared to 1064 nm Nd:YAG laser monotherapy.

## Author Contributions

**Zhang Jiang:** data curation (equal), formal analysis (equal), investigation (equal), and writing – original draft (lead). **Peng Jiang:** data curation (equal), formal analysis (equal), investigation (equal), and writing – original draft (equal). **Feifeng Ran:** data curation (equal), investigation (equal), and resources (equal). **Simin Li:** data curation (equal) and resources (equal). **Yuanyuan Xu:** data curation (equal) and resources (equal). **Yushuang Yang:** data curation (equal) and resources (equal). **Leifeng Liang:** data curation (equal), resources (equal), and supervision (supporting). **Li Yang:** data curation (equal), project administration (lead), supervision (lead), and writing – review and editing (lead). **Changyuan Wei:** conceptualization (lead), project administration (lead), supervision (lead), and writing – review and editing (lead).

## Funding

The authors have nothing to report.

## Ethics Statement

The study protocol received approval from the Ethics Committee of The First People's Hospital of Yulin, Guangxi (Approval No. YLSY‐IRB‐SR‐2026006).

## Conflicts of Interest

The authors declare no conflicts of interest.

## Data Availability

All raw data and code are available upon request.
